# Advances in pH‐Sensitive Luminescent Transition Metal Complexes for Theranostic Applications

**DOI:** 10.1002/asia.70635

**Published:** 2026-02-11

**Authors:** Bishnu Das, Sakira Tabassum Borah

**Affiliations:** ^1^ Department of Chemistry Ångström Laboratory Uppsala University Uppsala Sweden; ^2^ Department of Chemical Sciences Indian Institute of Science Education and Research Kolkata India

## Abstract

pH sensitive luminescent transition metal complexes are emerging as next generation molecular probes for sensing, bioimaging, and cancer theranostics. Small variations in protonation state can markedly influence their photophysical behavior by modulating charge‐transfer processes, excited‐state energetics, and noncovalent interactions, resulting in pronounced and often reversible changes in emission intensity or wavelength. In this review, we examine recent advances in the design of pH‐responsive luminescent transition metal complexes and discuss the mechanistic origins of their pH sensitivity, including protonation‐deprotonation equilibria, hydrogen‐bonding effects, and aggregation‐induced phenomena. Particular emphasis is placed on ruthenium(II) and related complexes, which offer long‐lived excited states, structural robustness, and wide tunability through rational ligand design. We critically analyze representative systems across different metal platforms with respect to their pK_a_ ranges, emission responses, and suitability for biological environments. By highlighting both recent progress and existing limitations, we aim to clarify design strategies that are most likely to translate into reliable tools for real‐time pH sensing, high‐resolution imaging, and multifunctional theranostic applications.

## Graphical abstract

This first comprehensive review unifies recent developments in pH‐sensitive luminescent transition metal complexes, outlining mechanistic insights that connect luminescence modulation with theranostic function and inspire future design strategies.

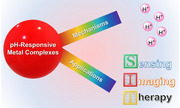

## 1 Introduction

Accurate pH sensing is crucial for understanding physiological and pathological processes within living systems, both at the intracellular and extracellular levels [1, 2, 3, 4]. Traditional pH indicators, though widely used, often suffer from limitations such as low sensitivity, narrow dynamic range, and poor biocompatibility, driving the development of metal‐based pH sensors with superior sensitivity, specificity, and stability [5]. Among these, luminescent transition metal complexes have gained significant attention due to their tunable photophysical properties and ability to monitor pH in complex biological environments.

**FIGURE 1:**
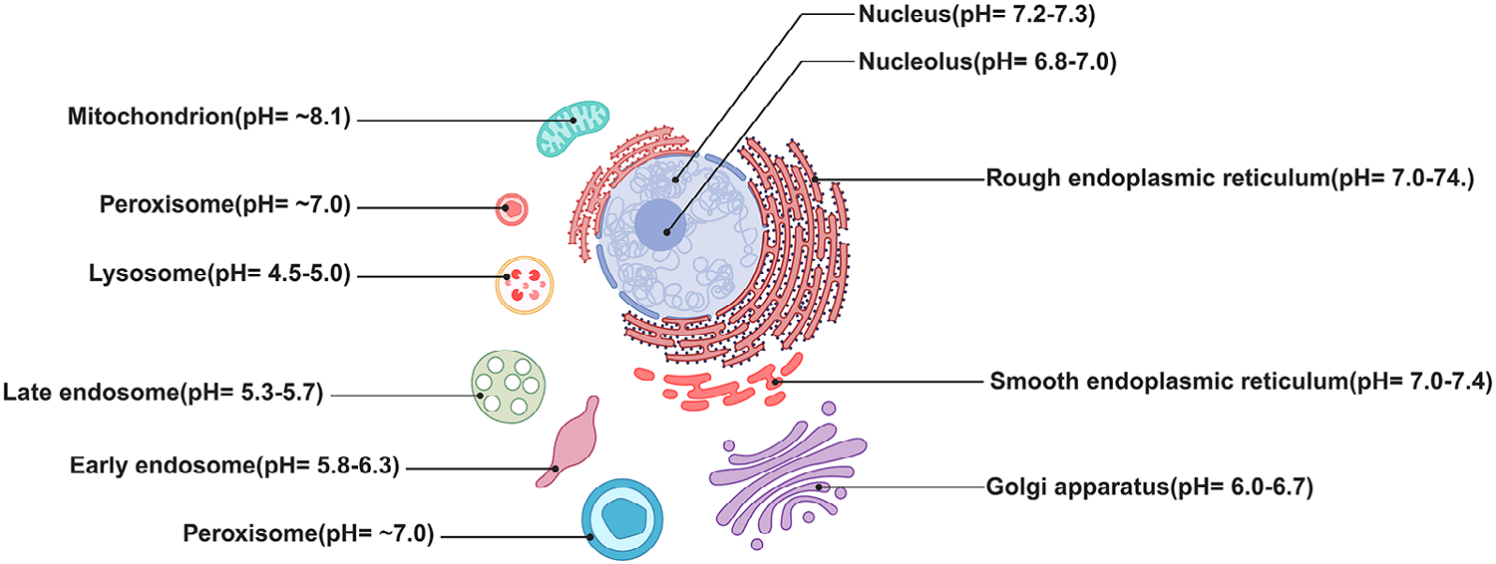
Illustration of cellular organelles with their respective pH levels. Created with Biorender.com.

The importance of precise pH monitoring is further highlighted by its biological relevance. Abnormal pH distributions are closely linked to disease, with acidic tumor microenvironments (pH ∼6.5‐6.9) contrasting sharply with normal extracellular pH (∼7.4) [6]. Similarly, organelles such as lysosomes (pH 4.5‐5.0) and mitochondria (pH ∼8.1) rely on tightly regulated pH to maintain their function, and disruptions contribute to disorders ranging from lysosomal storage diseases to neurodegeneration (Figure 1) [7, 8, 9, 10, 11]. Despite extensive progress, many existing pH probes fall short under physiological conditions, highlighting the need for more reliable systems suitable for real‐time imaging and diagnostics in both healthy and pathological environments.

To meet these biological challenges, luminescent transition metal complexes such as zinc(II), rhenium(I), ruthenium(II), osmium(II), iridium(III), and platinum(II) derivatives have emerged as powerful candidates. Their long‐lived excited states, tunable emission wavelengths, and environmental sensitivity make them particularly suitable for pH sensing in complex biological systems [12, 13, 14]. Understanding the mechanisms governing their pH responsive luminescence, particularly the influence of protonation, hydrogen‐bonding (H‐bonding), and aggregation, has enabled advances in organelle‐specific imaging, disease diagnostics, and theranostic applications.

In this review, we present a comprehensive overview of recent progress in pH‐sensitive luminescent transition metal complexes and the fundamental principles that govern their behavior. We first describe the key mechanistic pathways responsible for pH‐responsive luminescence, including protonation‐deprotonation, H‐bonding, and aggregation‐induced effects. We then highlight representative systems based on zinc(II), ruthenium(II), iridium(III), platinum(II), and mixed‐metal architectures, with emphasis on structure‐property relationships, pK_a_ ranges, and emission responses. Finally, we discuss emerging applications in intracellular pH imaging and targeted cancer theranostics, and outline current challenges and future opportunities.

## 2 Mechanism of pH‐Responsive Behavior

The pH dependent luminescence of metal complexes originates from protonation induced electronic and structural changes in their ligand frameworks [15, 16]. These changes modulate charge transfer processes such as MLCT (Metal‐to‐Ligand Charge Transfer) and ICT (Intramolecular Charge Transfer), altering emission intensity and wavelength [17, 18, 19]. H‐bonding can further regulate the excited state dynamics and influence the luminescence efficiency [20, 21, 22, 23, 24, 25]. In addition, aggregation can induce or enhance emission through restriction of intramolecular motion, giving rise to aggregation induced emission [26, 27]. Collectively, these effects explain why many metal complexes respond strongly to pH changes, although their sensitivity can vary significantly between chemical and biological environments.

### 2.1 Protonation‐Deprotonation of Ligand Groups

**FIGURE 2:**
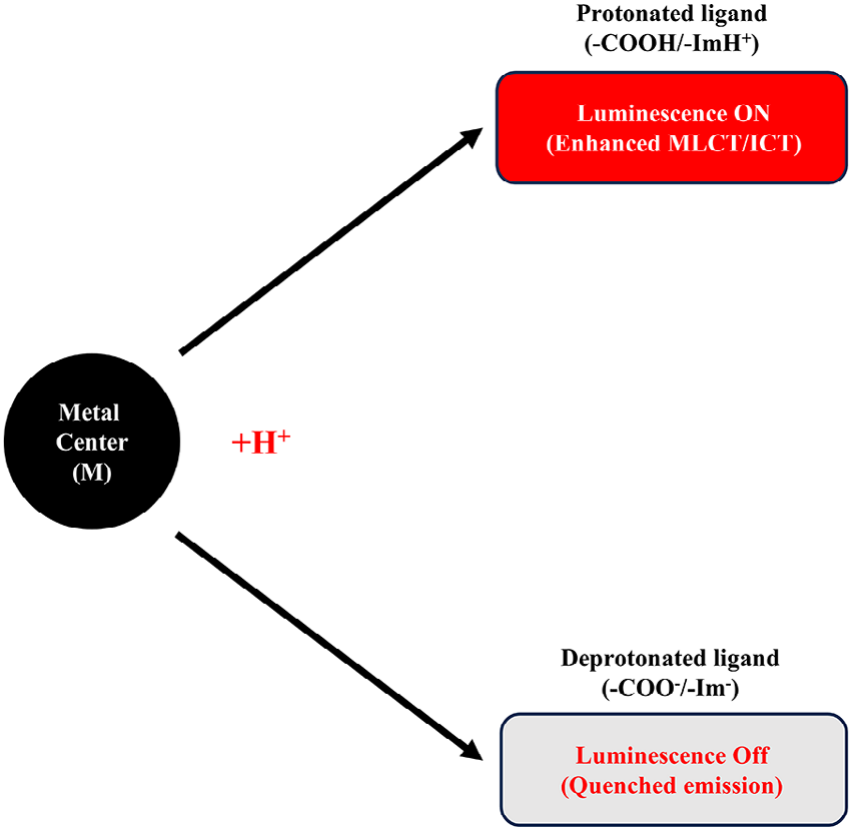
Schematic illustration of protonation‐deprotonation of ligand groups controlling luminescence switching.

A primary mechanism involves the protonation and deprotonation of ligand groups, which directly control the photophysical properties of metal complexes [28, 29]. Functional groups such as imidazole, carboxylate, and amide moieties undergo reversible protonation, reshaping the electronic environment of the metal center [30, 31]. At low pH, protonation of groups like imidazole or carboxylate alters their donor character, increasing electron density on the ligand atoms and thereby enhancing MLCT or ICT efficiency [32, 33]. This interplay between ligand protonation states and electronic structure underpins the pH sensitivity observed in many luminescent complexes, as schematically illustrated in Figure 2.

#### 2.1.1 Luminescence on/off Switching

pH‐responsive metal complexes can undergo luminescence on/off switching, governed by ligand protonation that modulates energy transfer and emission properties. Under neutral to slightly acidic conditions, protonation facilitates energy transfer to the metal center, producing strong luminescence (“on”), whereas highly acidic or basic environments cause quenching through altered oxidation potentials, excited‐state deactivation, or nonradiative pathways (“off”) [32, 33]. This switching behavior is particularly useful for monitoring pH fluctuations in biological systems.

Among such systems, ruthenium(II) complexes are especially attractive owing to their long‐lived excited states, high quantum yields, and strong MLCT characteristics. Their ligand frameworks can be systematically modified for tailored pH responsiveness, making them excellent candidates for luminescent pH sensors.

**FIGURE 3:**
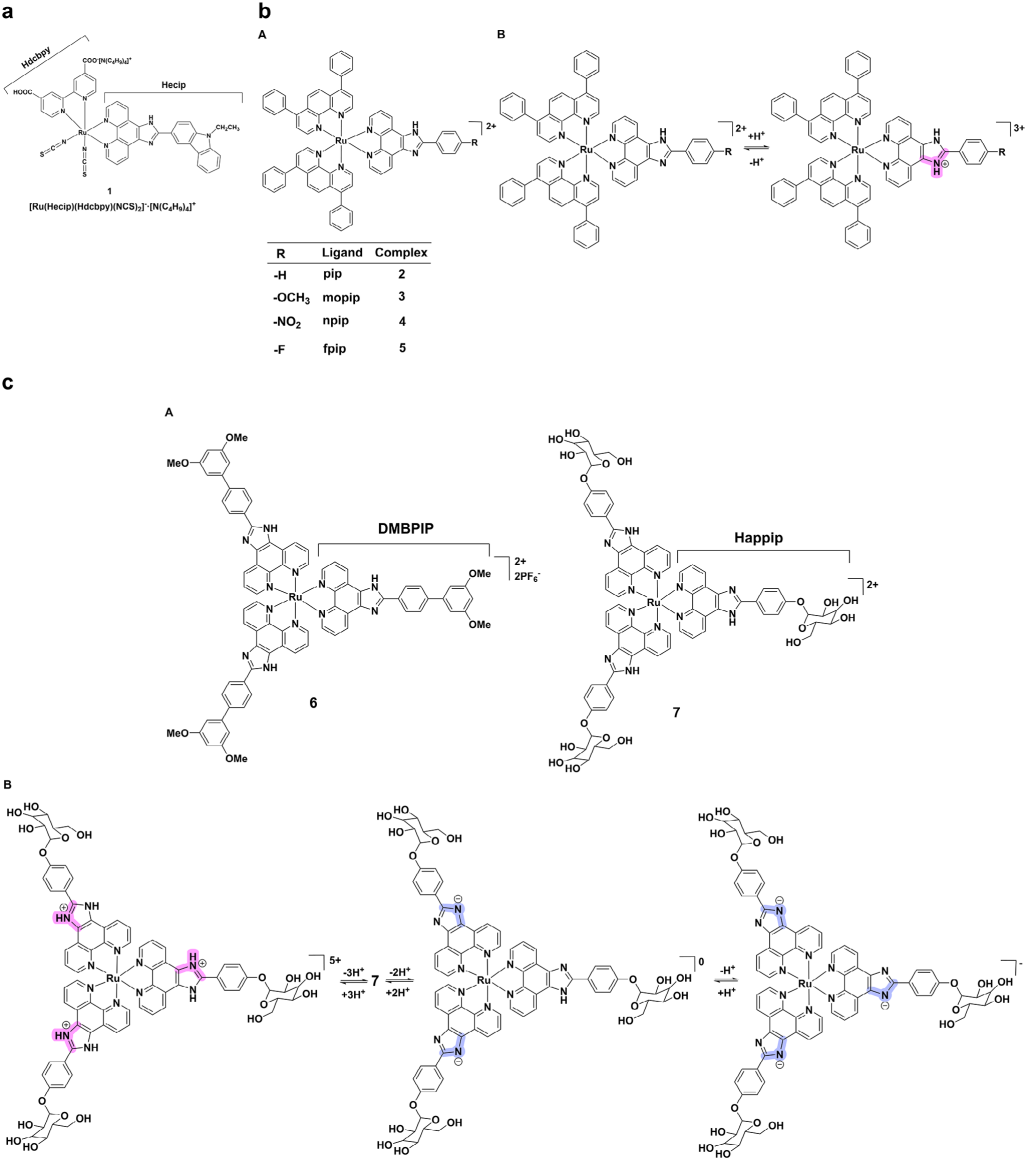
(a) Structure of complex **1**. (b) (A) Structures of complexes **2–5**; (B) Acid‐base equilibrium associated with complexes **2–5**. (c) (A) Structures of complexes **6** and **7**; (B) Acid‐base equilibrium corresponding to complex **7**.

Mononuclear ruthenium(II) complexes form the foundation for pH‐sensitive luminescent sensors, displaying well‐characterized protonation‐dependent switching [34]. Their precise emission modulation across pH ranges also provides a basis for more advanced architectures, including dinuclear and heterobimetallic systems. A representative example is complex **1** (Figure 3a), which shows “on‐off” switching between pH 2.1‐3.2 (enhanced luminescence via protonated carboxyls) and pH 8.0‐13.0 (quenched emission upon deprotonation) [35]. Complexes **2–5** (Figure 3b), featuring imidazole‐containing ligands, extend this concept; notably, complex **3** exhibits an exceptional on/off ratio of 110, attributed to electron donation by a methoxy substituent enhancing MLCT processes [36]. Further tunability is exemplified by complex **6** (Figure 3c), which undergoes two distinct luminescence transitions (off‐on at pH 2.0‐5.7 and on‐off at pH 5.7‐10.9), accompanied by emission wavelength shifts [37]. The broadest responsiveness is achieved with Ru(Happip)_3_^2+^ (**7**) (Figure 3c), which undergoes three successive protonation events (pK_a1_ = 1.44, pK_a2_ = 7.33, pK_a3_ = 12.20), yielding an unusual “on‐off‐on” switching pattern across the full pH range 0–13.7 [38]. In ruthenium(II) complexes, modest changes to ligand structure, including electron‐donating substituents or field‐tuning groups, are often sufficient to achieve precise and reversible modulation of luminescence.

**FIGURE 4:**
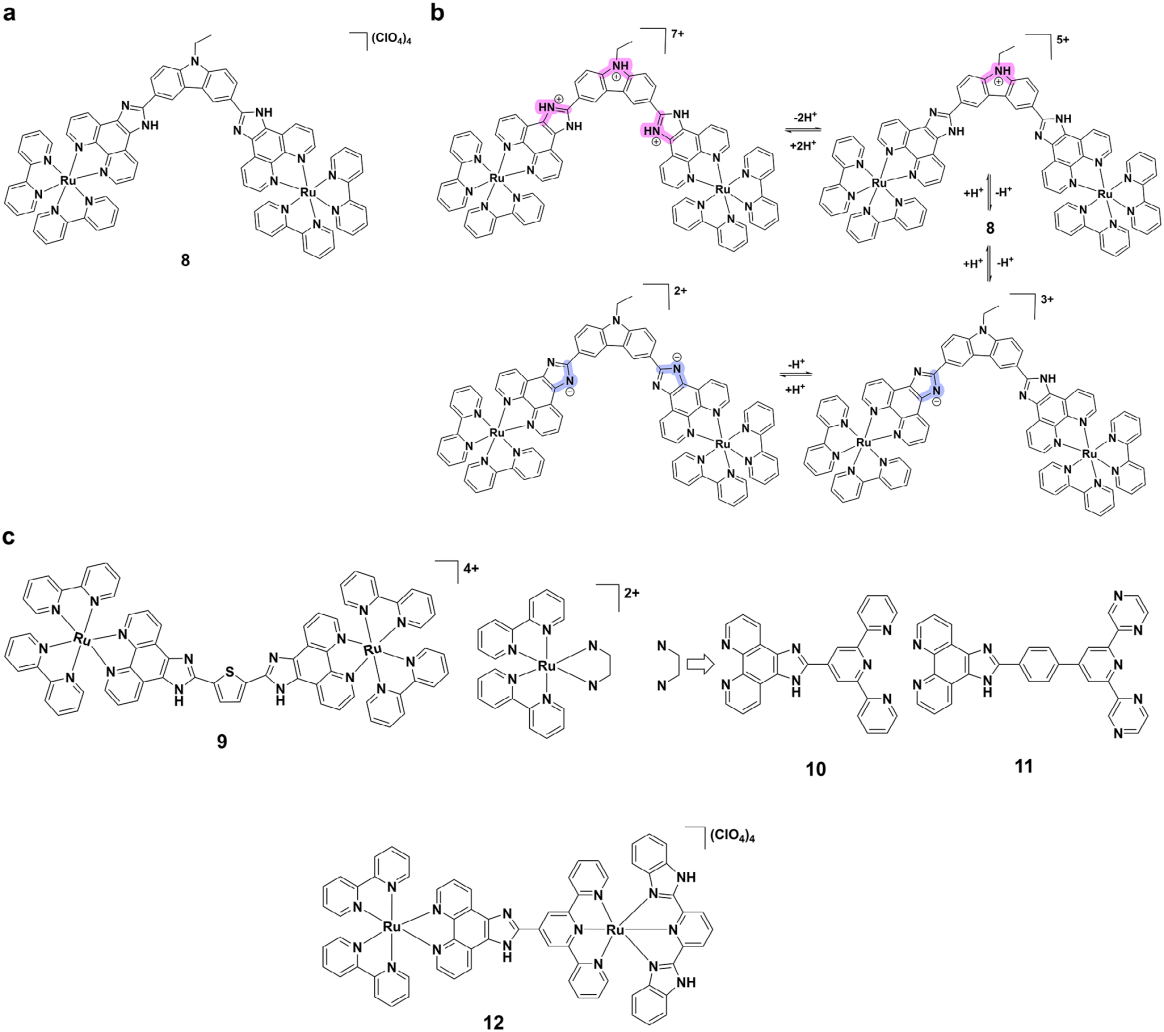
(a) Structure of complex **8**. (b) Acid‐base equilibria of complex **8**. (c) Structures of complexes **9–12**.

While mononuclear ruthenium(II) complexes establish the foundation for pH‐responsive luminescence, dinuclear systems offer enhanced sensitivity and broader tunability through multi‐step protonation‐dependent emission switching. Complex **8** (Figure 4a) operates as an efficient “on‐off” luminescence switch over pH 1.46‐12.80, with transitions at pH 4.16, 5.07, 9.65, and 12.09 arising from successive deprotonations of imidazole and carbazole groups. A pronounced quenching between pH 8.0‐10.0 yields an on/off ratio of ∼100, surpassing related imidazole‐containing systems (Figure 4b) [39]. Complex **9** (Figure 4c) exhibits an unusual “off‐on‐off” switching at 610 nm across pH 0.1‐13.2, governed by three protonation/deprotonation steps of the H_2_bipt ligand. At very acidic pH (0.1‐2.2), protonated imidazole rings boost luminescence 40‐fold, followed by progressive quenching upon deprotonation, highlighting its potential for monitoring pH in complex environments [40]. Complex **10** (Figure 4c) demonstrates a reversible “off‐on‐off” response over pH 1.0‐11.4. Protonation of imidazole and terpyridyl groups quenches luminescence at low pH, while deprotonation of pyridyl groups near pH 5.44 enhances emission 166‐fold. Further deprotonation of imidazole groups induces partial quenching with a red shift, accompanied by a 37.5‐fold increase in lifetime from acidic to neutral pH. [41] Similarly, complex **11** (Figure 4c) functions as a pH‐sensitive switch between pH 1.0‐11.0. Protonation of imidazole groups quenches emission, while deprotonation around pH 5.40 restores it; subsequent steps again reduce intensity, with excited‐state pK_a_* values of 5.29 and 7.98 indicating enhanced basicity [42]. Further expanding functionality, complex **12** (Figure 4c) displays a unique NIR “off‐on‐off” switching in BR buffer, emitting at 760 nm with a quantum yield of 1.04% and a lifetime of 108.3 ns. Luminescence is enhanced at pH 1.0‐3.5, quenched at 7.0‐11.0, and governed by protonation/deprotonation of imidazole and benzimidazole groups (pK_a_ = 1.36, 5.76, 9.01). This tunability highlights its promise in bioimaging and cancer diagnostics [43].

Dinuclear ruthenium(II) complexes go beyond their mononuclear counterparts by providing multi‐state emission control, sharper switching, and wider pH responsiveness. The combination of cooperative metal‐ligand interactions and precisely tuned protonation sites allows these systems to exhibit highly dynamic and reversible luminescence, making them strong candidates for real‐time cellular imaging, targeted diagnostics, and multifunctional molecular devices.

Beyond homonuclear and dinuclear ruthenium(II) systems, mixed‐metal architectures introduce additional electronic interactions that expand the tunability of pH‐responsive luminescence [18]. Introducing a secondary metal center, such as osmium(II), expands spectral diversity and enables multi‐state emission switching, making these complexes promising candidates for advanced photochemical sensing and imaging applications.

**FIGURE 5:**
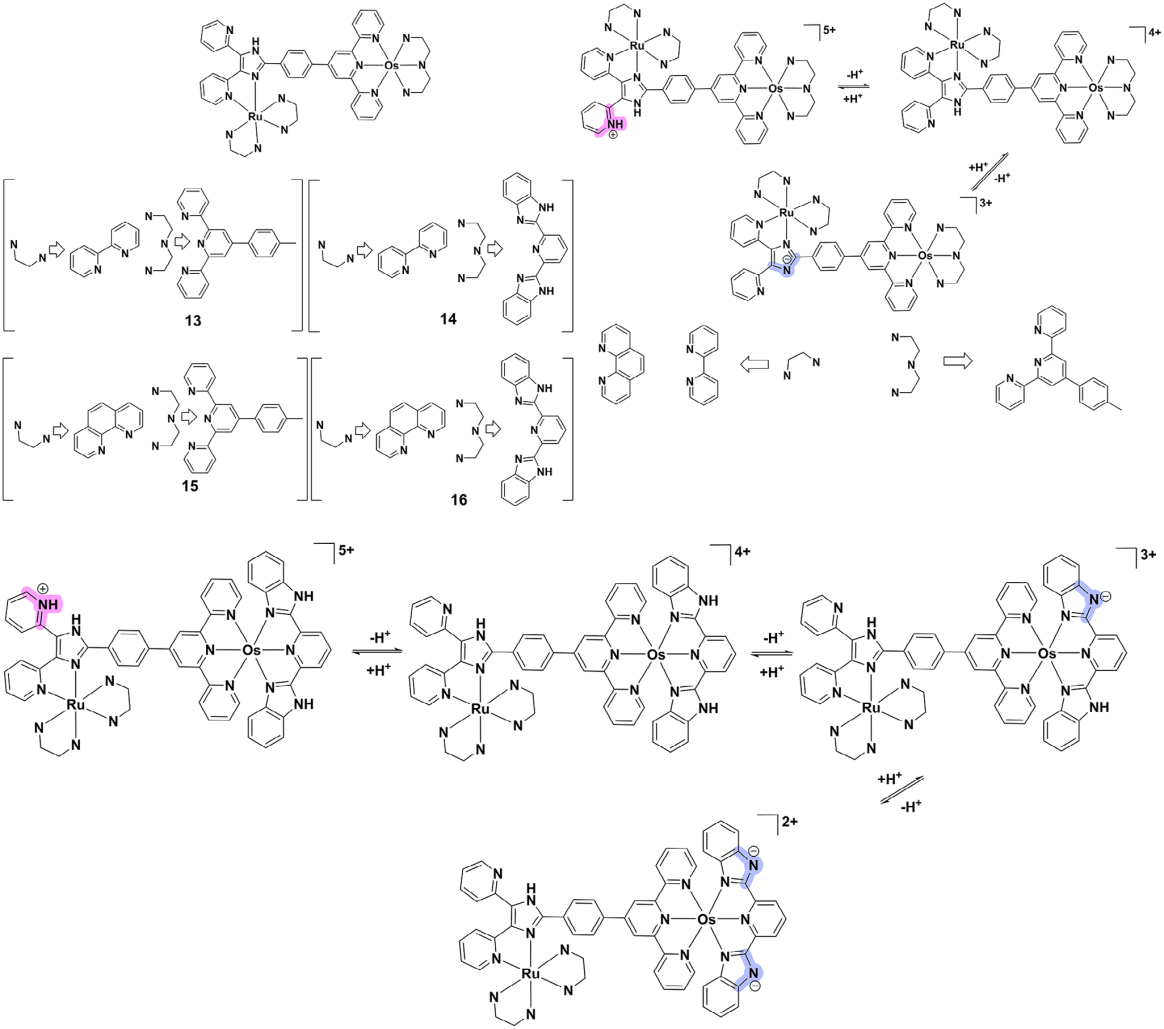
Structures and acid‐base equilibria of complexes **13–16**.

The ruthenium(II)‐osmium(II) heterobimetallic complex **13** (Figure 5) exemplifies this strategy, displaying a two‐step reversible luminescence switch controlled by protonation‐deprotonation of pendant imidazole groups in the secondary coordination sphere [44]. While the primary coordination sphere consists of terpyridine/bipyridine ligands directly chelating the metal centers, the imidazole groups, though not directly bound, modulate the photophysical properties through H‐bonding and electrostatic effects. Protonation at low pH quenches the luminescence (“on‐off”), while deprotonation restores intensity upon return to neutral/basic conditions. This reversible switching emphasizes the importance of secondary‐sphere interactions in fine‐tuning optical outputs. Complex **14** (Figure 5) exhibits analogous two‐state switching but with subtle differences in intensity modulation, arising from variations in the protonation states of imidazole groups and their influence on intramolecular Ru(II)→Os(II) energy transfer. While the switching profile resembles that of complex **13**, the enhanced robustness of complex **14** suggests potential utility in environments requiring more stringent stability [44]. A more intricate response is observed in complex **15** (Figure 5), which undergoes three‐step emission switching as pH varies. Here, multiple protonation‐deprotonation equilibria in the imidazole rings yield distinct luminescence states, producing a finely tunable multistate system [44]. Such multi‐step control offers significant advantages for applications demanding precision modulation of optical signals. Similarly, complex **16** (Figure 5) displays three‐state switching, but with stronger coupling to Ru(II)→Os(II) photoinduced intramolecular energy transfer [44]. Proton dissociation events in the imidazole groups directly alter energy‐transfer efficiency, thereby modulating emission intensity. This distinctive multi‐step behavior makes complex **16** a promising candidate for pH‐responsive bioimaging and phototherapy applications.

Hence, ruthenium(II)‐osmium(II) heterobimetallic complexes exemplify how mixed‐metal systems can be engineered for sophisticated pH‐sensitive behavior. Their capacity for reversible “on‐off” or multi‐step luminescence switching arises from the interplay of protonation‐driven coordination changes and secondary‐sphere‐controlled energy transfer. These design principles highlight the versatility of mixed‐metal complexes as next‐generation tools for near‐infrared imaging, real‐time sensing, and multifunctional phototherapy.

#### 2.1.2 Luminescence Intensity Modulation

Luminescence on/off switching is a valuable tool for probing pH fluctuations in biological systems, where precision is critical [45, 46, 47]. However, many systems respond in a graded rather than binary manner, with emission intensity varying continuously with protonation state. Such luminescence intensity modulation provides nuanced optical readouts, where protonation/deprotonation events tune emission intensity rather than fully quenching or activating it. In pH‐responsive metal complexes, modulation arises from ligand protonation states, which alter the electronic environment of the metal center and thereby impact excited‐state energy transfer and emissive pathways. Protonation generally increases electron density and boosts emission, whereas deprotonation can open nonradiative decay channels, reducing luminescence. This behavior underpins the development of reversible, sensitive pH sensors. A diverse range of complexes including zinc(II), ruthenium(II), iridium(III), platinum(II), and heterometallic systems, exhibit such intensity‐based modulation, governed by electronic communication between metal centers and protonatable ligands.

Zinc(II) complexes, owing to their closed‐shell d^10^ configuration, lack metal‐centered transitions, and display luminescence dominated by ligand‐centered excited states [48]. This renders their photophysics particularly sensitive to protonation‐induced orbital modulation, making them excellent candidates for precise pH‐responsive luminescence control. Their nontoxic and redox‐inert nature further supports biological applications.

**FIGURE 6:**
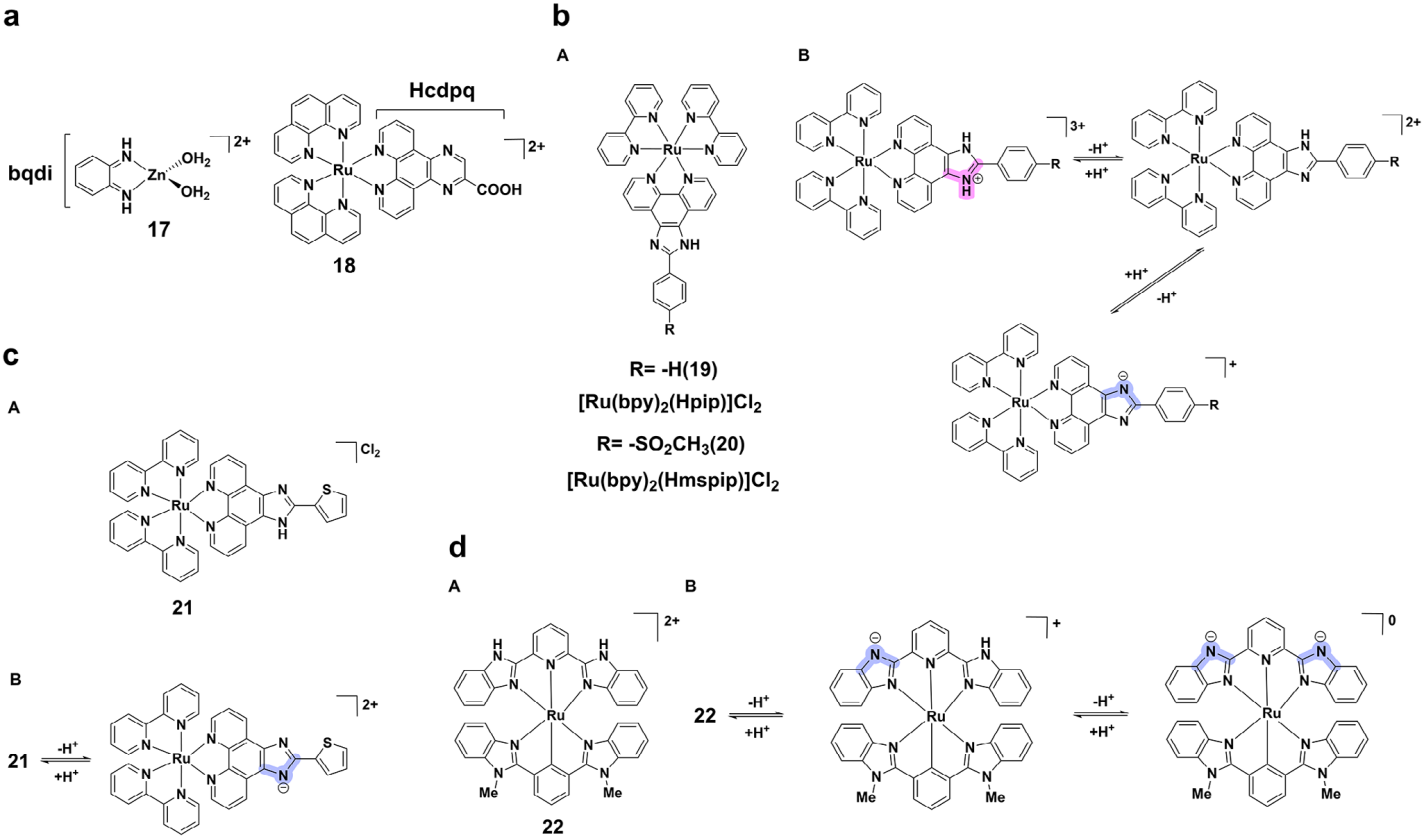
(a) Structures of complexes **17** and **18**. (b) (A) Structures of complexes **19** and **20**; (B) Acid‐base equilibria of complexes **19** and **20**. (c) (A) Structure of complex **21**; (B) Acid‐base equilibria of complex **21**. (d) (A) Structure of complex **22**; (B) Two successive proton‐transfer equilibria of complex **22**.

A notable example is complex **17** (Figure 6a), which exhibits strong visible emission at 576 nm (Φ = 0.026, τ = 1.6 ns) at pH 8.0, but shows an eightfold decrease in intensity and a red‐shift to 638 nm (τ = 131 ps) at pH 3.0 [49]. These changes correlate with the reversible protonation/deprotonation of the bqdi ligand (pK_a_ ≈ 5.2), supported by isosbestic points in the absorption spectra. Such reversible luminescence modulation highlights the potential of zinc(II) complexes as turn‐on fluorescent probes with finely tunable output.

Ruthenium(II) complexes, with long‐lived MLCT excited states, high photostability, and favorable redox properties, are especially promising for pH‐responsive luminescence in aqueous/physiological media [50]. Their emission is readily modulated via ligand protonation, enabling applications in molecular switches, imaging probes, and energy‐related systems.

Mononuclear examples include complex **18** (Figure 6a), where protonation of the carboxyl group on Hcdpq suppresses luminescence at pH<1.8, but deprotonation enhances intensity 17‐fold at neutral pH (pK_a_ = 3.06 ± 0.08) [51]. Complexes **19** and **20** (Figure 6b), incorporating imidazole‐based ligands, show two ionization events (pK_a1_ = 2.17, 1.16; pK_a2_ = 8.82, 8.05, respectively), with protonation‐driven MLCT absorption shifts and emission maxima shifting from 611 to 620 nm for complex **20** [52]. While complexes **18–20** structurally similar to complexes **2‐5** (Figure 3b), these systems operate through more gradual electronic changes at multiple protonation sites, which results in continuous intensity modulation instead of abrupt on/off switching. Similarly, complex **21** (Figure 6c) undergoes two proton dissociation steps (pK_a_ = 1.55, 7.3), with luminescence intensity reduced by 52% and a blue‐shift in emission from 625 to 615 nm as pH increases, reflecting both ground‐ and excited‐state acidity constants [53]. Complex **22** (Figure 6d) illustrates proton‐coupled electron transfer (PCET), with benzimidazole ligands showing pK_a1_ = 3.50 and pK_a2_ = 5.83 in the Ru(III) state, producing pH‐dependent absorption red‐shifts relevant to redox‐battery applications [54].

**FIGURE 7:**
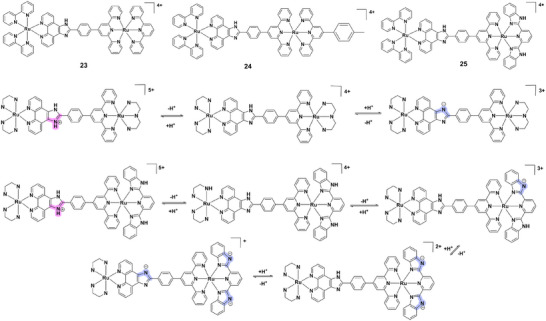
Structures and acid‐base equilibria of complexes **23–25**.

**FIGURE 8:**
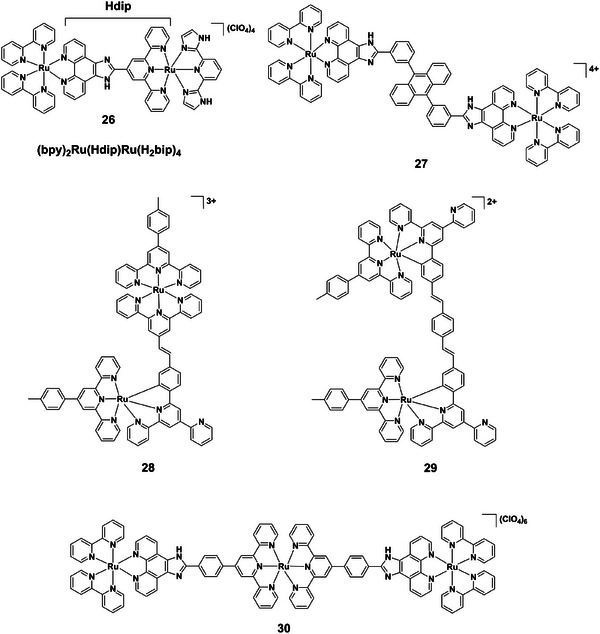
Structures of complexes **26–30**.

Dinuclear ruthenium(II) complexes enhance this tunability through cooperative effects. Complexes **23–25** (Figure 7) exhibit successive deprotonation between pH 2.5‐12.0, showing enhanced luminescence in acidic media and quenching in alkaline conditions [55]. Complex **26** (Figure 8) operates reversibly in the physiological pH window (6.0‐8.0), displaying a large Stokes shift (∼200 nm), strong near‐IR emission (∼700 nm), excellent photostability, and negligible cytotoxicity (IC_50_> 100 µM), highlighting its bioimaging potential [56]. Binuclear complex **27** (Figure 8), bearing imidazole ligands, displays red shifts, and reduced luminescence intensity with deprotonation across pH 1.81‐10.38, with ground‐ and excited‐state pK_a_ values of 3.46 and 7.15, respectively [57]. Further, ruthenium(II) terpyridine dimers **28** and **29** (Figure 8) show reversible luminescence quenching upon pyridyl protonation, accompanied by red‐shifts and accelerated photo‐isomerization under acidic conditions, demonstrating molecular switch functionality [58].

Trinuclear ruthenium(II) complexes extend these concepts, with greater electronic communication across three centers. For instance, heteroleptic complex **30** (Figure 8) undergoes sequential imidazole deprotonation (pH 2–4 and 8–10), with absorption changes at 496, 400, 284, and 334 nm and clear isosbestic points [59]. Luminescence intensity is enhanced between pH 2–7 (factor 2.54), but quenched at higher pH, accompanied by emission red‐shifts. Excited‐state pK_a_* values exceed ground‐state pK_a_, reflecting altered basicity and excited‐state localization.

In mono‐, di‐, and trinuclear forms, ruthenium(II) complexes convert protonation‐induced electronic perturbations into tunable luminescence responses. While these systems have been explored in physiological sensing, photo‐responsive devices, and redox‐active platforms, their versatility is not uniform and remains closely tied to structural design.

Iridium(III) complexes stand out among transition‐metal luminophores due to their efficient phosphorescence, which originates from strong spin‐orbit coupling and high triplet quantum yields [60, 61, 62, 63, 64]. Their emission is highly tunable through protonation‐induced modulation of MLCT and ligand‐centered states. In contrast to zinc(II) or ruthenium(II) systems, iridium(III) complexes combine excellent photostability with long excited‐state lifetimes, rendering them powerful candidates for time‐resolved pH sensing and phosphorescence lifetime imaging microscopy (PLIM) in biological settings. Bis‐terpyridine and cyclometalated derivatives are especially attractive because of their robust photophysical properties. Protonation of their ligands alters electronic structures, thereby modulating emission intensity, lifetime, and spectral output. This pH‐responsive behavior has been widely explored in both bis‐terpyridine and cyclometalated iridium(III) complexes, highlighting their potential in optical pH sensing and biological imaging [65, 66, 67, 68].

**FIGURE 9:**
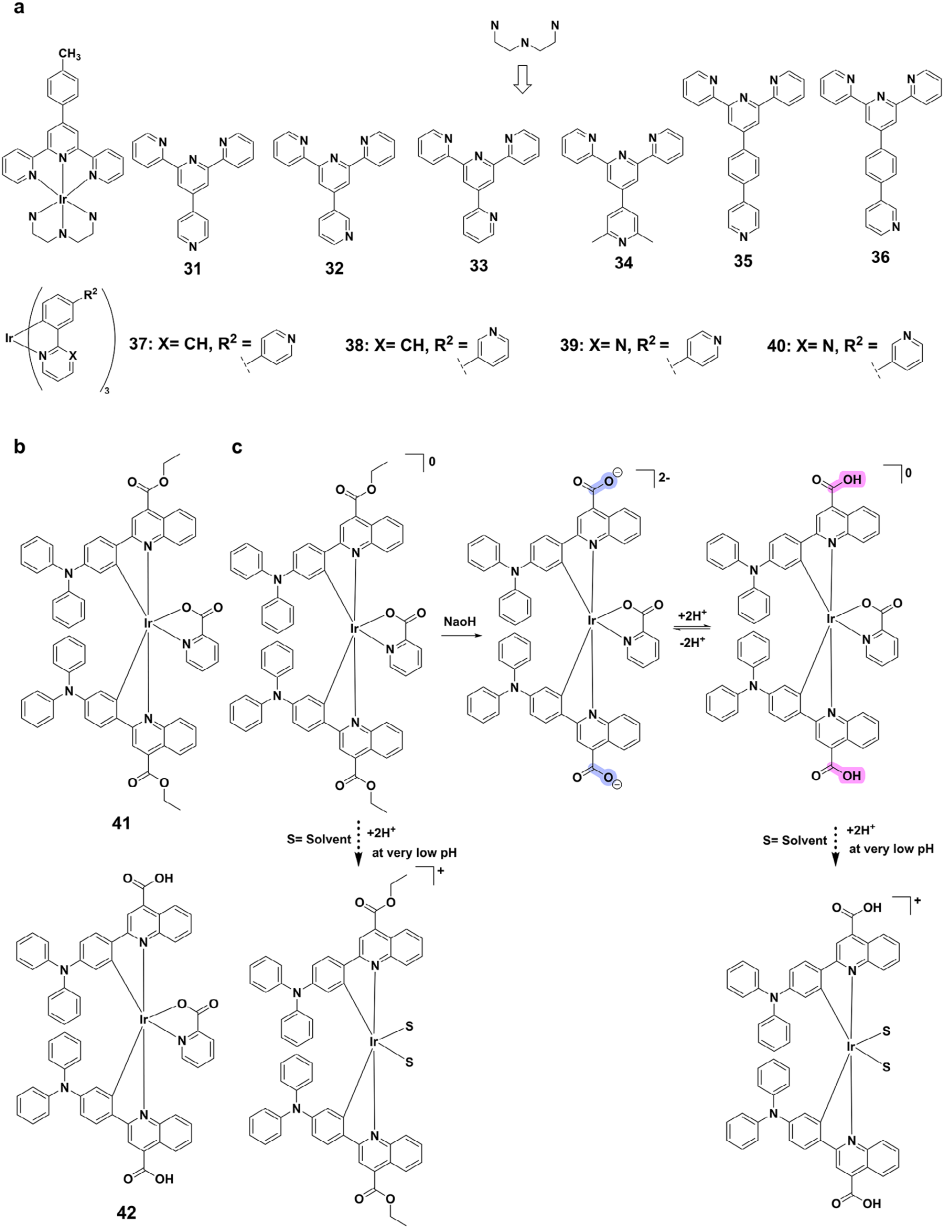
(a) Structures of complexes **31–40**. (b) Structures of complexes **41** and **42**. (c) Schematic representation of the proposed equilibrium and reactions explaining the effects of alkali and acid on complexes **41** and **42**.

A representative example is the series of iridium(III) bis‐terpyridine complexes **31–36** (Figure 9a) [69]. Complexes **31**, **33**, and **34**, bearing para‐ and ortho‐pyridyl substituents, undergo strong emission quenching with ∼eightfold lifetime shortening. Complex **33** displays an inflection point shifted to lower pH, while the meta‐pyridyl analogue **32** remains pH‐insensitive, underlining the importance of ortho/para configurations. The 2,6‐dimethylpyridyl derivative **34**, with enhanced basicity, shifts its response to higher pH. Mechanistically, protonation lowers higher‐lying MLCT states, promoting nonradiative decay and shorter lifetimes. Complexes **35** and **36** further diversify this behavior; specifically, protonation induces blue shifts in their emission spectra, with complex **36** showing particularly strong wavelength sensitivity, offering opportunities for wavelength‐encoded pH probes. Iridium(III) complexes **37–40** (Figure 9a) illustrate complementary pH‐driven luminescence modulation [70]. Complex **37** emits green light (∼498 nm) at neutral/basic pH but red‐shifts to ∼600 nm with weakened intensity under acidic conditions (pH 4–5). Similar intensity enhancement without major spectral shift is observed for complex **38**, while complex **39** switches from weak orange emission at low pH to strong yellow emission at high pH. Importantly, the ground‐ and excited‐state pK_a_^*^ values differ significantly (e.g. for complex **37**), evidencing distinct protonation mechanisms. Such tunable spectral and intensity changes underpin their utility in pH sensing.

**FIGURE 10:**
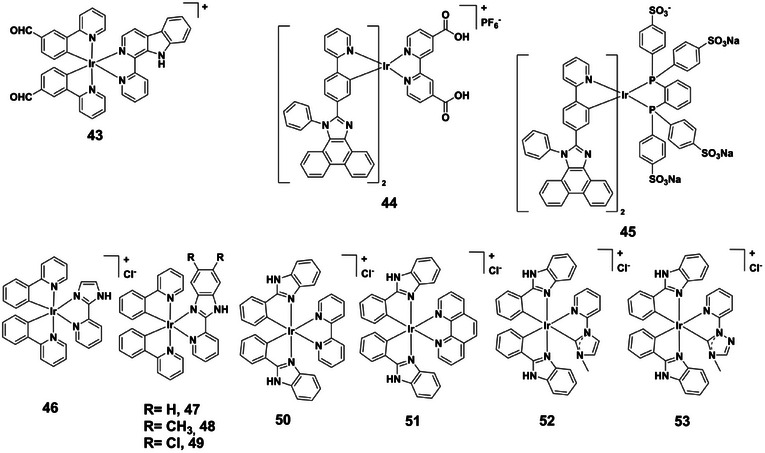
Structures of complexes **43–53**.

Cyclometalated complexes **41** and **42** (Figure 9b) further showcase ligand‐dependent pH responsiveness [71]. Complex **42**, containing a carboxyl group, undergoes deprotonation under basic conditions, yielding a blue‐shifted MLCT absorption band and higher quantum yield, with a near‐linear emission response across pH 6.5‐8.0. In contrast, ester‐functionalized complex **41** shows minimal response, while complex **42** also red‐shifts and quenches under acidic conditions (Figure 9c). These traits make complex **42** particularly promising for aqueous and bioprocessing environments. Complex **43** (Figure 10) exemplifies pronounced protonation‐controlled emission [72]. At pH 2.2 it emits intense yellow‐green light (540 nm), but intensity decreases sharply with increasing pH, becoming nearly non‐emissive above pH 6.2. The calculated pK_a_ (3.86) confirms protonation of ligand donor sites, which stabilizes the excited state and suppresses nonradiative relaxation. Protonation thus enhances quantum yields and lifetimes, while deprotonation accelerates quenching. Emission lifetimes are further reduced under aerated conditions due to oxygen quenching, which also enhances singlet oxygen generation at low pH. This makes complex **43** highly suitable for probing acidic microenvironments. Complexes **44** and **45** (Figure 10) demonstrate contrasting pH‐dependent responses [73]. Complex **44** undergoes a bathochromic shift from 600 to 650 nm upon protonation, driven by a transition from mixed ^3^LC/^3^MLCT to ^3^LLCT/^3^MLCT states, with a sigmoidal lifetime‐pH profile (pK_a_ ≈ 6.2). Complex **45** retains a broad 560 nm band in both protonated and deprotonated forms, but shows enhanced intensity and lifetimes at lower pH (pK_a_ ≈ 4.15). Together, they illustrate protonation‐induced modulation of excited‐state configurations and lifetimes, offering direct relevance for PLIM‐based intracellular pH monitoring. Finally, iridium(III) complexes **46–53** (Figure 10), incorporating imidazole or benzimidazole ligands, highlight substituent and ancillary‐ligand effects on pH responsiveness [74]. Complexes **46–49** show blue‐shifted emission (24‐85 nm) and intensity enhancement upon increasing pH, whereas complexes **50–53** undergo quenching or red‐shifts. Their pK_a_ values span 5.19‐11.22, reflecting how electron‐donating/withdrawing substituents and ancillary ligands (e.g. NHCs, pyridyl‐imidazolylidenes, pyridyl‐triazolylidenes) modulate basicity. This behavior arises from ligand protonation/deprotonation, altering HOMO‐LUMO transitions and luminescence output.

Iridium(III) complexes, especially bis‐terpyridine and cyclometalated derivatives, show rich pH‐responsive photophysics driven by ligand protonation. Their emission can shift from green (∼498 nm) to red (∼650 nm), vary in intensity, and change in lifetime, typically with pK_a_ values between 3.5 and 8.5. Combined with long phosphorescence lifetimes, strong photostability, and tunable wavelengths, these complexes are highly versatile for real‐time pH sensing in aqueous and biological systems, including live‐cell PLIM.

While iridium(III) complexes exhibit remarkable pH dependent photophysics driven by ligand protonation, platinum(II) complexes expand this functionality through distinct excited state processes and coordination geometries [75, 76, 77, 78]. In their monomeric form, platinum(II) complexes primarily display conventional MLCT and ligand centered transitions. However, upon aggregation through Pt···Pt interactions, triplet metal‐metal‐to‐ligand charge transfer (^3^MMLCT) states can emerge, significantly influencing their emission behavior and pH responsiveness. Their square planar geometry facilitates strong π‐π stacking and Pt···Pt interactions, often giving rise to aggregation induced emission and halochromic responses. These characteristics underpin their use as smart, reversible pH sensors with both colorimetric and luminescent outputs.

**FIGURE 11:**
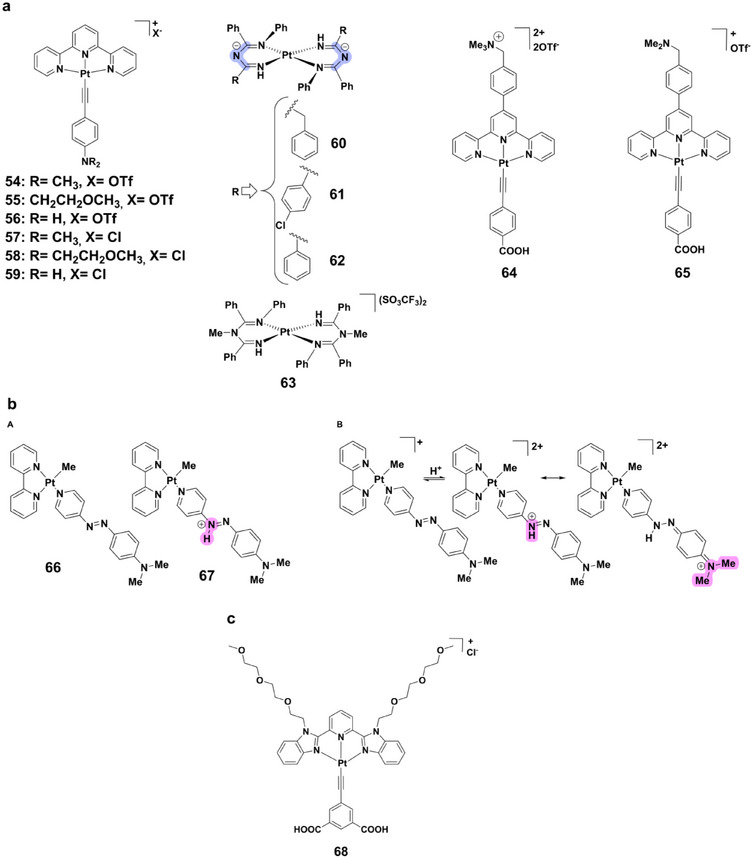
(a) Structures of complexes **54–65**. (b) (A) Structures of complexes **66–67**; (B) Protonation mechanism of complex **66**. (**c**) Structure of complex **68**.

A prominent class of pH‐responsive platinum(II) complexes is the terpyridyl‐alkynyl derivatives **54–59** (Figure 11a) [79]. Protonation of their amino substituents decreases the electron‐donating strength of the alkynyl ligands, producing a blue shift in the low‐energy LLCT (Ligand‐to‐Ligand Charge Transfer) absorption band and enhancing emission at 560–563 nm. These changes are accompanied by dramatic color transitions from purple to yellow, enabling naked‐eye pH sensing. Their pK_a_ values reflect substituent effects, with complex **57** exhibiting the highest pK_a_ due to electron‐donating methyl groups.

Structurally distinct imidoylamidinate derivatives **60–62** (Figure 11a) display unique protonation‐dependent emission [80]. Protonation of the central nitrogen atom quenches luminescence completely in acidic media, while basic conditions restore strong emission near 500 nm (quantum yields: 3.7×10^−4^ to 6.2×10^−2^ in methanol). This response is reversible upon neutralization. The quenching mechanism arises from protonation‐induced sp^2^→sp^3^ rehybridization, which disrupts conjugation and activates nonradiative d‐d pathways. The bis‐protonated model complex **63** confirmed this, showing no luminescence in either solution or solid state.

Water‐soluble alkynyl‐platinum(II) terpyridine complexes **64** and **65** (Figure 11a) further highlight the interplay of protonation and self‐assembly [81]. Protonation of carboxyl groups (pK_a_* = 2.51 for complex **64**) or deprotonation of ‐CH_2_NMe_2_ substituents (pK_a_* = 3.26 and 8.53 for complex **65**) modulates hydrophilicity, controlling aggregation. At low pH, enhanced Pt···Pt/π‐π interactions promote strong near‐infrared ^3^MMLCT emission, whereas deprotonation at higher pH increases hydrophilicity, disperses aggregates, and reduces luminescence via photoinduced electron transfer (PET) quenching.

Beyond terpyridyl frameworks, azo‐ligand‐based platinum(II) complex **66** (Figure 11b) exhibits a striking halochromic effect [82]. Protonation converts complex **66** into complex **67**, shifting the MLCT/π‐π* absorption band from 433 to 540 nm, with a clear isosbestic point at 474 nm. In dichloromethane, a similar red shift occurs (495→598 nm). The process is fully reversible: deprotonation with triethylamine restores the original spectrum. The absence of photoisomerization in protonated complex **67** suggests structural stabilization, making this system highly attractive for reversible colorimetric pH sensing.

Expanding functional versatility, platinum(II) complex **68** (Figure 11c) demonstrates protonation‐induced charge reversal, enabling self‐assembly with polyelectrolytes such as PFP‐OSO_3_^−^ [83]. This assembly enhances Förster resonance energy transfer from the polyelectrolyte to complex **68**, generating pronounced ^3^MMLCT emission in the red region. The ensemble exhibits strong ratiometric luminescence changes with excellent sensitivity and selectivity. Importantly, it retains its reversible, photostable response in diluted human and bovine serum, with minimal interference from biomacromolecules, demonstrating clear biological applicability.

Platinum(II) complexes illustrate how square‐planar coordination and accessible excited states can be exploited to achieve pH‐responsive behavior. Depending on ligand design, these systems can display luminescent or colorimetric responses, aggregation‐controlled emission, and reversible protonation‐driven switching. Importantly, reported pK_a_ values span acidic to basic regimes, enabling real‐time, and reversible pH monitoring under biologically relevant conditions.

While platinum(II) complexes have shown remarkable pH‐responsive luminescence arising from protonation‐induced modulation of charge‐transfer states and aggregation effects, mixed metal complexes offer a complementary strategy by incorporating multiple metal centers with distinct and synergistic photophysical properties [84]. Systems combining ruthenium(II), rhodium(III), iridium(III), and rhenium(I) are particularly attractive, as intermetallic charge transfer processes and cooperative ligand effects provide enhanced tunability of luminescence intensity, spectral shifts, and excited‐state lifetimes. This interplay of multiple centers often leads to more pronounced and switchable luminescence responses compared to single‐metal systems, making them promising candidates for sensing, bioimaging, and molecular electronics.

**FIGURE 12:**
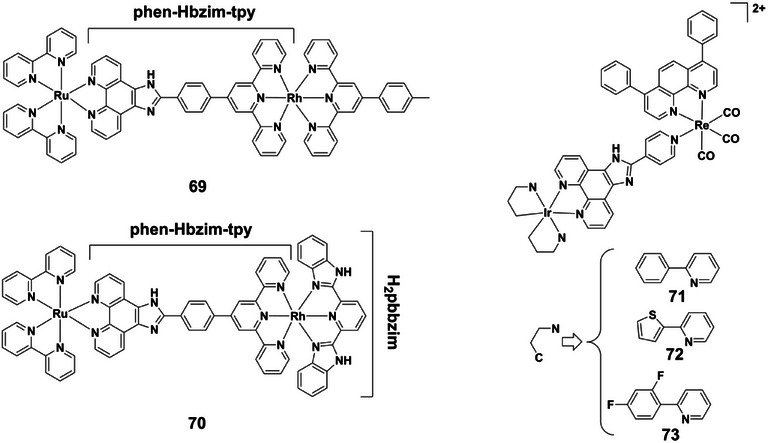
Structures of complexes **69–73**.

Representative homo‐ and heterobimetallic complexes **69** and **70** (Figure 12) illustrate this principle, employing a heteroditopic phenanthroline‐terpyridine bridge functionalized with proton‐responsive imidazole N‐H groups (phen‐Hbzim‐tpy) and an H_2_pbbzim moiety [55]. Spectrophotometric and luminescence titrations across pH 2.5‐12 revealed two to four distinct deprotonation steps, each correlating with changes in absorption and emission. Protonation at acidic pH (2.5‐7.5) enhances luminescence, while higher pH values (7.5‐12) reduce intensity, particularly in ruthenium‐based homobimetallic complexes. Protonation also induced red shifts in the MLCT bands, underscoring the potential of these systems as proton‐driven molecular switches for photonic and electronic applications. Further extending this approach, heterobimetallic iridium(III)‐rhenium(I) complexes **71–73** (Figure 12) exhibit dramatic pH‐responsive luminescence modulation [85]. Protonation of N‐H groups on the imidazole rings reversibly enhances emission, with acidic conditions (pH 2.4) leading to ∼sevenfold (**71**), 17‐fold (**72**), and 19‐fold (**73**) intensity increases compared to neutral or alkaline media (pH 7.4), where emission is weak or effectively quenched. This pronounced “off‐on” switching behavior makes them particularly well suited for tumor cell imaging in acidic intracellular environments such as lysosomes and endosomes. Their additional cytotoxicity further highlights potential as lysosome‐targeting anticancer agents.

In mixed‐metal complexes, the interplay between pH‐responsive ligands and metal‐metal interactions offers additional routes to tuning luminescence. While these cooperative effects allow precise control over photophysical behavior, their practical utility depends on maintaining stability and reproducibility across different application contexts.

### 2.2 Hydrogen‐Bonding

Beyond direct protonation‐deprotonation effects on ligand charge distribution and electronic states, H‐bonding interactions introduce an additional level of control over the photophysics of metal complexes. H‐bonds act as secondary‐sphere interactions that subtly modify local electronic environments and redistribute electron density within ligand frameworks, thereby influencing charge‐transfer processes and excited‐state energetics [86]. By stabilizing charge‐separated excited states and lowering excited‐state reorganization energies, H‐bonding can prolong excited‐state lifetimes and promote radiative decay, leading to enhanced luminescence efficiency. Moreover, H‐bond‐induced perturbations of excited‐state potential energy surfaces can suppress nonradiative relaxation pathways such as internal conversion and vibrational dissipation, while in some systems H‐bonding is also closely associated with excited‐state proton transfer or PCET processes that significantly influence excited‐state relaxation dynamics and emission behavior [87, 88].

**FIGURE 13:**
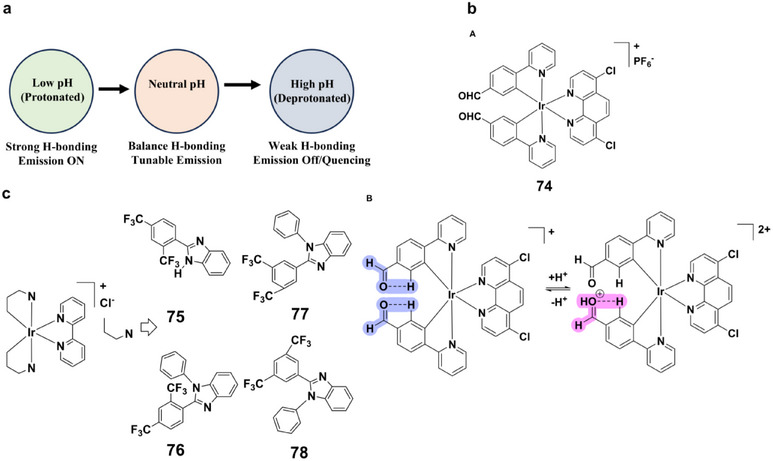
(a) Schematic diagram illustrating the effect of pH on H‐bonding and emission. (b) (A) Structure of complex **74**; (B) Proposed pH‐responsive mechanism for probe **74** involving intramolecular H‐bonding. (c) Structures of complexes **75–78**.

Within this framework, protonation can strengthen or establish new H‐bonding networks, thereby modulating charge‐transfer processes and luminescence, while deprotonation can weaken or disrupt such interactions, altering excited‐state lifetimes and emission intensities (Figure 13a). This dynamic interplay between protonation states and H‐bonding enables precise tuning of luminescence, highlighting H‐bonding as an important consideration in the design of pH‐responsive materials.

An aldehyde‐bearing iridium(III) complex **74** (Figure 13b) exemplifies this concept, designed to monitor intracellular pH, a parameter critical in physiological and pathological processes [89]. Complex **74** displayed strong pH‐dependent luminescence between pH 1.81 and 6.81, with a pK_a_ of 4.30. At lower pH, luminescence decreased slightly and blue‐shifted, attributed to a H‐bond‐assisted mechanism in which protonation perturbed the H‐bonding network. The complex further offered rapid response, high photostability, low cytotoxicity, and excellent cell permeability, while selectively localizing to mitochondria in HeLa cells. Real‐time imaging showed that complex **74** can track mitochondrial pH changes during PMA‐induced apoptosis, revealing early‐stage mitochondrial acidification and highlighting its potential as a sensitive biomedical imaging tool.

Expanding this strategy, complexes **75–78** (Figure 13c) highlight how ligand design governs H‐bonding and thereby pH responsiveness [90]. Derived from isomeric bis‐trifluoromethyl‐substituted benzimidazole ligands, these complexes revealed pronounced differences in photophysics depending on the availability of intramolecular H‐bonds. Complex **75** exhibited strong intramolecular H‐bonding, stabilized by metal coordination and geometric rigidity, resulting in enhanced luminescence under acidic conditions (pH 1–6). By contrast, N‐Ph‐substituted analogues **76** and **78** lacked imidazolyl N‐H groups and thus failed to establish such H‐bonding networks, leading to diminished pH responsiveness. These comparisons clearly demonstrated how H‐bonding can be strategically exploited to fine‐tune charge‐transfer dynamics and pH sensitivity. Notably, the robust pH response of complex **75** points to potential applications in sensing biogenic amines, where precise control of luminescence switching is essential.

These studies show that H‐bonding can strongly influence charge‐transfer pathways and emission behavior. When carefully integrated into ligand frameworks, H‐bonding motifs offer a flexible means of tuning pH‐responsive luminescence for sensing and bioimaging applications.

### 2.3 Aggregation‐Induced Effect

**FIGURE 14:**
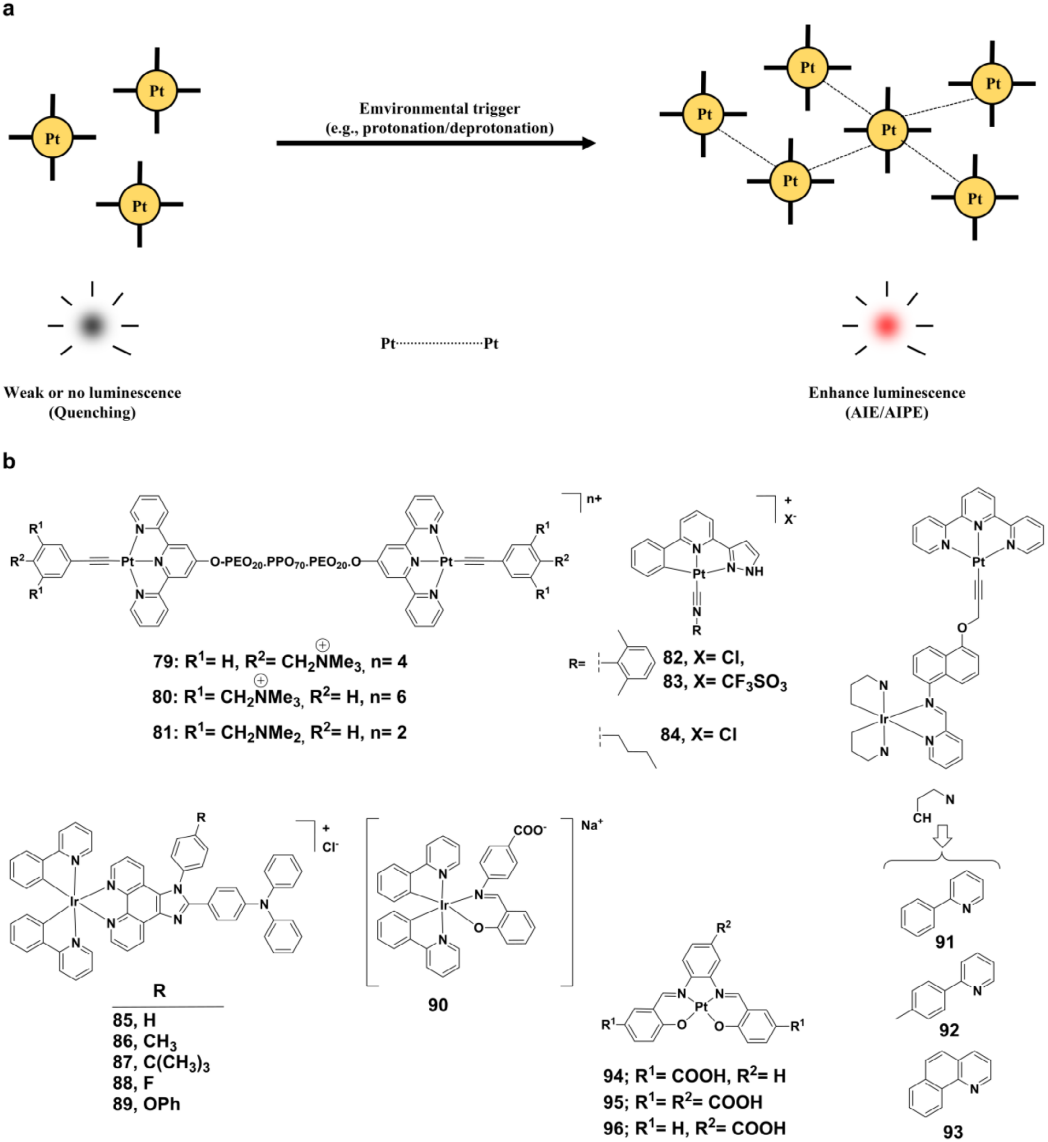
(a) Schematic diagram of aggregation induced effect; (b) Structures of complexes **79–96**.

Beyond H‐bonding, aggregation‐induced emission (AIE) offers another powerful strategy for achieving pH‐responsive luminescence (Figure 14a) [91]. Unlike conventional fluorophores prone to aggregation‐caused quenching, AIE‐active systems display enhanced luminescence upon aggregation [92, 93, 94, 95]. This behavior originates from restricted intramolecular rotations and strengthened π‐π interactions, which suppress nonradiative decay pathways and favor radiative emission. Owing to their dynamic luminescence response to environmental changes, AIE‐active metal complexes have emerged as promising candidates for bioimaging, cancer therapy, and pH sensing.

Platinum(II) and iridium(III) complexes constitute particularly versatile AIE‐active platforms. Terpyridyl platinum(II)‐based triblock copolymers **79–81** (Figure 14b) demonstrate reversible pH‐ and temperature‐dependent micellization, where Pt⋅⋅⋅Pt and π‐π interactions induce significant near‐infrared (NIR) emission changes [96]. Among these, complex **81**, containing CH_2_NMe_2_ substituents, shows dual‐responsive NIR luminescence that arises from ligand protonation/deprotonation coupled with aggregation. In another series, platinum(II) complexes **82–84** (Figure 14b) undergo pH‐driven self‐assembly through protonation of the pyrazole NH group [97]. This induces a striking emission shift from green to orange, facilitating selective lysosomal accumulation, membrane disruption, and subsequent cell death, key attributes for targeted cancer therapy. Complex **82** further exhibits hydrogel‐forming ability, enabling sustained therapeutic release and dual‐function behavior as a drug delivery and anticancer agent.

Iridium(III) complexes extend AIE‐based strategies to real‐time biological imaging. Triphenylamine‐appended complexes **85–89** (Figure 14b), incorporating 2‐phenylimidazo[4,5‐f][1,10]phenanthroline ligands, exhibit remarkable photostability and minimal photobleaching, allowing long‐term monitoring of mitochondrial processes such as mitophagy [98]. A water‐soluble monoanionic iridium(III) complex **90** (Figure 14b) displays finely tuned pH‐responsive AIE behavior: at pH 4.7, protonation triggers aggregation, producing enhanced phosphorescence and a bathochromic emission shift (508→ 618 nm), while extreme acidity (pH < 2.8) induces dissociation and emission quenching [99]. Such dual modulation highlights its suitability for precise bioimaging within physiologically relevant pH windows.

Heterometallic systems further expand the AIE toolkit. Iridium(III)‐platinum(II) complexes **91–93** (Figure 14b) exhibit aggregation‐induced phosphorescence (AIPE) under acidic conditions, with emission maxima at 660 nm (**92**) and 685 nm (**93**) [100]. Dynamic light scattering confirmed reversible aggregation governed by pH and ionic strength, underscoring their potential for selective bacterial imaging in acidic microenvironments. Similarly, platinum(II) salophen complexes **94–96** (Figure 14b), bearing carboxyl substituents, exhibit reversible AIE enhancement through protonation/deprotonation of carboxyl groups [101]. Aggregation in aqueous mixtures produces strong visible emission, tunable via the both number and position of carboxyl units, making these systems excellent candidates for advanced pH‐sensing applications.

AIE provides a practical strategy for the design of luminescent pH sensors. Examples ranging from NIR‐emissive platinum(II) assemblies to photostable iridium(III) complexes and heterometallic AIPE‐active systems illustrate how aggregation can support stable, reversible, and finely tunable luminescence responses. By mitigating photobleaching, extending imaging durations, and improving environmental stability, AIE‐active complexes have been explored for applications in cancer therapy, microbial diagnostics, mitochondrial imaging, and environmental monitoring.

As a whole, the mechanisms discussed above demonstrate how protonation‐deprotonation equilibria, H‐bonding, and aggregation phenomena collectively govern the pH‐responsive luminescence of transition metal complexes. These mechanistic pathways lead to distinct photophysical behaviors across different metal systems, influencing their pK_a_ ranges, emission profiles, and functional performance in sensing and biomedical applications. To provide a comparative overview, the key characteristics of pH‐sensitive luminescent metal complexes discussed in this review are summarized in Table 1.

**TABLE 1:** Inter‐system comparison of pH‐sensitive luminescent metal complexes.

Metal system	Typical pK_a_ range	Luminescence response	Emission characteristics	Representative applications
Zn(II)	∼3.0‐8.0	Gradual intensity modulation	Ligand‐centered fluorescence (visible); short lifetimes (ps‐ns)	Intracellular pH sensing, low‐toxicity fluorescent probes, photodynamic/photothermal therapy
Ru(II)	∼1.0‐13.7	On/off, multi‐state switching, or graded modulation	MLCT‐based emission (visible‐NIR); long lifetimes (ns‐µs)	Cellular and organelle pH imaging, molecular switches, theranostics
Ir(III)	∼3.5‐11.2	Intensity modulation and wavelength shifts	Strong phosphorescence (green‐red); long triplet lifetimes	PLIM imaging, lysosomal and mitochondrial pH mapping, theranostics
Pt(II)	∼2.0‐9.0	On/off switching, aggregation‐enhanced emission	MLCT/LLCT and ^3^MMLCT emission (visible‐NIR)	Colorimetric pH sensing, lysosomal imaging, self‐assembled theranostics
Mixed‐metal systems (Ru‐Os, Ir‐Re, Ir‐Pt, Ru‐Ru)	Broad (≈ 2.0‐12.0)	Multi‐step and amplified luminescence responses	Dual‐ or multi‐channel emission	Ratiometric imaging, pH logic gates, multifunctional theranostics

## 3 Applications of pH‐Sensitive Metal Complexes

The versatile photophysical responses of pH‐sensitive metal complexes underpin their widespread application in biomedical imaging and therapy. Protonation‐deprotonation events modulate their charge‐transfer and electronic states, enabling precise pH sensing, high‐resolution imaging, and even targeted therapeutic action. Key areas include intracellular imaging of organelle‐specific pH fluctuations and the design of smart pH‐responsive agents for selective cancer therapy.

### 3.1 Intracellular pH Imaging

Mapping intracellular pH is crucial for understanding cellular homeostasis, since organelles such as lysosomes, mitochondria, and endosomes operate under distinct acidic or near‐neutral environments essential for their function [102, 103, 104, 105, 106]. Dysregulation of these pH gradients is closely linked to cancer, neurodegeneration, and metabolic disorders [107, 108, 109]. To address this, luminescent metal‐based probes have been developed with high sensitivity, specificity, and stability, enabling real‐time tracking of intracellular pH dynamics in live cells. Among these, iridium(III) complexes stand out for their tunable emission, photostability, and low cytotoxicity.

**FIGURE 15:**
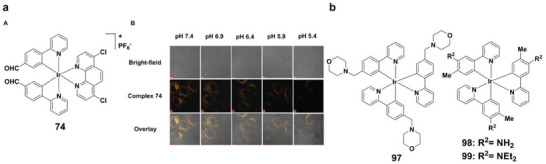
(a) (A) Structure of complex **74**; (B) CLSM images of HeLa cells at varying pH levels. HeLa cells were treated with 20 µM complex **74** for 30 min, fixed with 4% formaldehyde, and exposed to fresh cell culture medium (without phenol red) at different pH values for 20 min. CLSM images were captured in the 550–630 nm range with 405 nm excitation. Scale bar = 5 µm. Reproduced with permission from [ref [89]]. Copyright 2023. Elsevier. (b) Structures of complexes **97–99**.

The first breakthroughs came from iridium(III) complexes **44** and **45** (Figure 10), which exhibited pH‐dependent phosphorescence and selectively localized in lysosomes [73]. Although their low quantum yield and oxygen sensitivity limited use, they provided valuable early insights into lysosomal pH fluctuations. A major advancement followed with aldehyde‐bearing complex **74** (Figures 13b and 15a), which exhibited reversible protonation‐controlled luminescence over a broad pH window (1.81‐6.81, pK_a_ = 4.30) [89]. Complex **74** combined photostability, low cytotoxicity, and rapid response, enabling real‐time monitoring of mitochondrial activity and apoptosis. Building on this, complexes **75–78**(Figure 13c), incorporating bis‐trifluoromethyl benzimidazole ligands, showed enhanced pH sensitivity. Among them, complex **75** gave strong emission enhancement in the acidic range (pH 4–6), ideal for lysosomal imaging and autophagy studies [90]. More recently, the water‐soluble tris‐cyclometalated complex **97** (Figure 15b) demonstrated stable emission across both acidic and alkaline ranges, with two‐photon excitation capability for long‐term tracking of cell migration and apoptosis [110].

Iridium(III) probes have also been extended to mitochondrial imaging. Complexes **85–8**9 (Figure 14b) track mitophagy with stable pH‐dependent luminescence across pH 4.0‐8.0, aided by triphenylamine units that enhance photostability [98]. Dual‐function probes such as **98** and **99** (Figure 15b) combine sensing and therapy. Notably, complex **99** not only monitors lysosomal pH but also generates singlet oxygen under irradiation in a pH‐dependent manner, enabling photoinduced necrosis‐like cell death, an integrated diagnostic and therapeutic platform for cancer [111].

Platinum(II)‐based probes further enrich intracellular pH imaging. Alkynylplatinum(II) terpyridines **64** and **65** (Figure 11a) undergo pH‐responsive self‐assembly, with near‐infrared emission that enhances organelle differentiation [81]. Complexes **82–84** (Figure 14b) exhibit dramatic color shifts from green to orange under acidic conditions (pH ∼4‐5), mimicking lysosomal environments [97]. Complex **82** also forms pH‐responsive hydrogels, integrating pH imaging with drug delivery.

Mixed‐metal systems expand this design space. Heterobimetallic iridium(III)‐rhenium(I) complexes **71–73** (Figure 12) show strong emission enhancement at lysosomal pH (∼2.4), offering selective cancer cell imaging [85]. Similarly, heterobimetallic iridium(III)‐platinum(II) complexes **91–93** (Figure 14b) exploit aggregation‐induced photoluminescence (AIPE) [100]. Complex **93** in particular demonstrates pH‐independent imaging at pH 5–6.8 due to enhanced π‐π stacking, enabling reliable tracking across diverse acidic microenvironments.

Iridium(III), platinum(II), and heterobimetallic complexes have been widely applied as molecular tools for intracellular pH mapping. Their photostability, organelle selectivity, and potential for multifunctionality, including combined sensing and therapeutic activity, have enabled detailed studies of autophagy, mitophagy, and cancer‐related processes.

### 3.2 Targeted Cancer Therapy

pH‐responsive metal complexes have emerged as powerful tools not only for imaging but also for selective cancer therapy. The acidic tumor microenvironment, arising from altered metabolism and hypoxia, provides an intrinsic trigger for tumor‐specific drug activation [112, 113, 114, 115, 116, 117]. Exploiting the acidic tumor microenvironment as a trigger for tumor‑selective activation has inspired numerous pH‑responsive designs. However, a broader range of metal complexes (including zinc(II), ruthenium(II), iridium(III), platinum(II), and mixed‑metal systems) have been engineered to combine therapeutic and imaging functions through diverse mechanisms, including but not limited to pH activation, enhancing tumor selectivity while minimizing systemic toxicity [118, 119, 120, 121, 122, 123, 124, 125, 126, 127, 128, 129].

**FIGURE 16:**
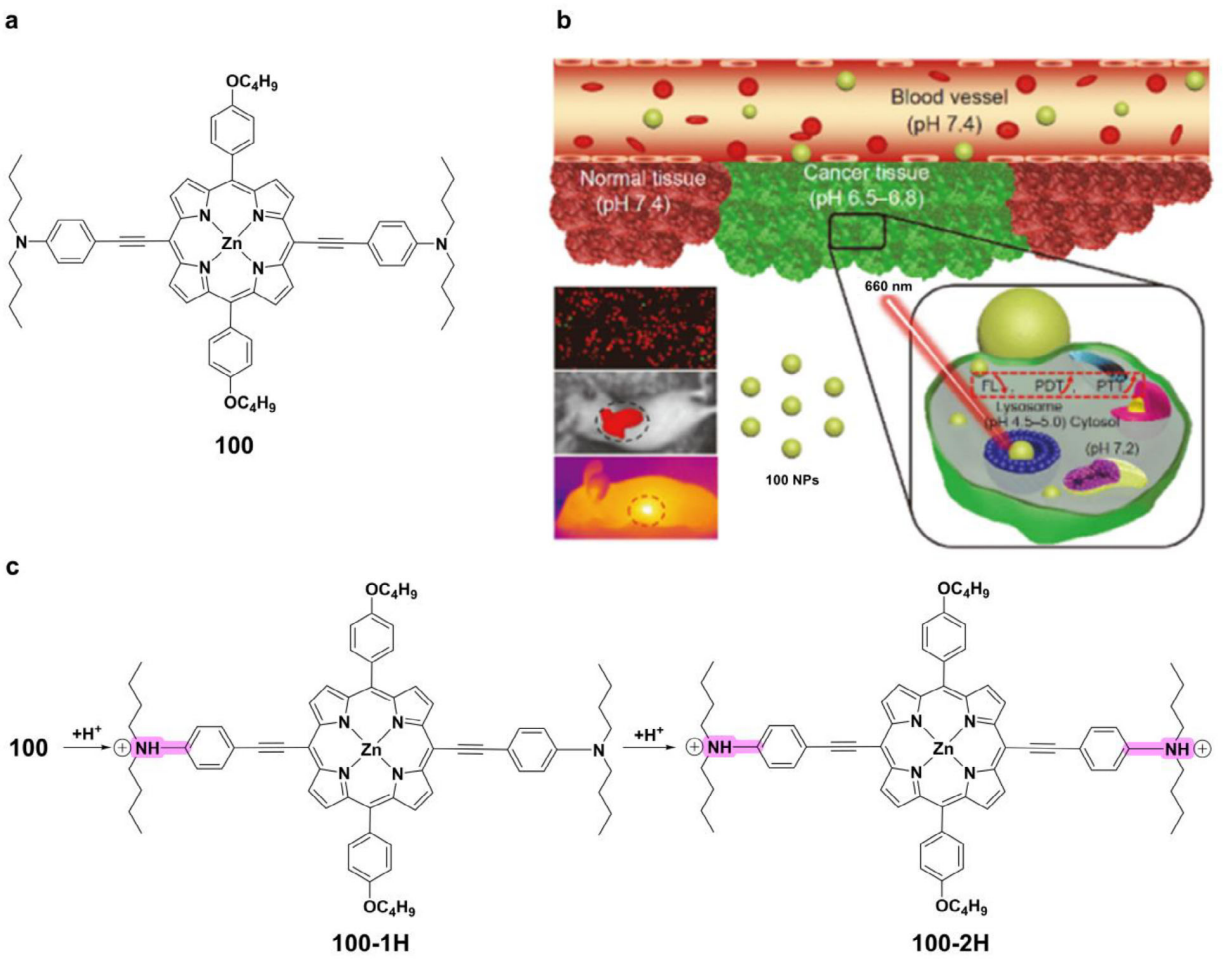
(a) Structure of complex **100**. (b) Schematic illustration of pH‐responsive cancer photothermal/photodynamic therapy guided by multi‐modal imaging using **100** nanoparticles in both *in vitro* and *in vivo* settings. Reproduced with permission from [ref [133]. Copyright 2019. Springer Nature. (c) Suggested mechanism of complex **100** protonation induced by acidic (H^+^) conditions.

Zinc(II)‐based complexes have shown particular promise in photothermal and photodynamic therapy [130, 131, 132]. A striking example is complex **100** (Figure 16a), a zinc(II)‐metalated porphyrin bearing N,N‐dibutyl‐4‐ethynylaniline (DBAP) units [133]. Protonation under acidic conditions yields mono‐ and diprotonated species (**100‐1H**, **100–2H**) with enhanced electronic absorption and efficient photothermal conversion (Figure 16b,c). As nanoparticles, complex **100** accumulates in lysosomes and mediates tumor ablation via combined photothermal/photodynamic effects, with high phototoxicity, negligible dark toxicity, and excellent stability, key features for next‐generation therapy.

**FIGURE 17:**
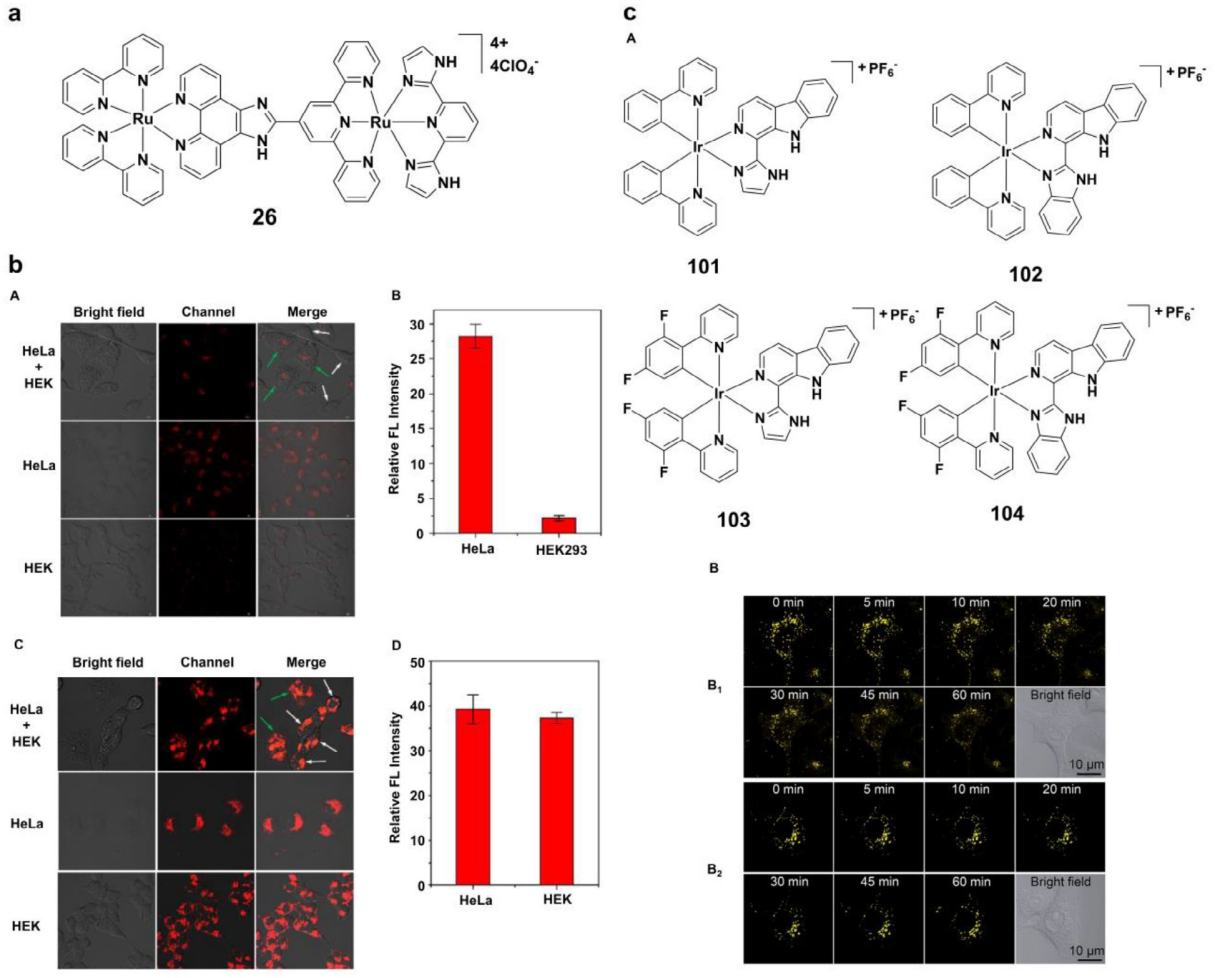
(a) Structure of complex **26**. (b) Comparison of cell imaging (A, C) and emission intensity bar graphs (B, D) for HeLa cells and healthy HEK293 cells incubated with complex **26** (left panel) and [Ru(bpy)_2_dtdpq](ClO_4_)_2_(right panel). Reproduced with permission from Ref. [56]. Copyright 2020. American Chemical Society. (c) (A) Structures of complexes **101–104**; (B) Real‐time monitoring of lysosomal integrity in A549 cells loaded with complex **102** (10 µM, 12 h) (B_1_) with or (B_2_) without photodynamic therapy treatment (120 s, 4.8 Jcm^−2^). Reproduced with permission from Ref. [144]. Copyright 2015. The Royal Society of Chemistry.

Ruthenium(II) complexes extend this concept by combining pH‐sensitive luminescence with tumor selectivity [134, 135, 136]. Complex **26** (Figures 8 and 17a) accumulates preferentially in HeLa cells, where its red luminescence is ∼13‐fold stronger than in HEK293 cells after 8 h incubation (Figure 17b) [56, 137]. This selectivity, absent in a non pH‐responsive analogue Ru(bpy)_2_dtdpq_2_, highlights the value of pH‐triggered uptake for distinguishing malignant from healthy cells, enabling both imaging and therapy.

Iridium(III) complexes leverage dual mechanisms: mitochondrial targeting and reactive oxygen species (ROS) generation [138, 139, 140, 141, 142, 143]. Complexes **37** and **38** (Figure 9a) show pH‐dependent emission shifts and efficient singlet oxygen production under neutral to basic conditions, inducing necrosis‐like death in cancer cells upon photoirradiation [70]. Cyclometalated complex **43** (Figure 10) exhibits high singlet oxygen generation in acidic environments, selectively killing HeLa cells while sparing normal cells [72]. Further innovations include β‐carboline derivatives **101–104** (Figure 17c), which combine lysosome‐specific imaging with pH‐triggered ROS production. Complex **102** achieved a phototoxicity index >833 against A549 cells, inducing apoptosis through lysosomal disruption [144]. In pH‐sensitive iridium(III) complexes, luminescence reporting and therapeutic ROS generation can be coupled with organelle targeting, although these functions are not always achieved with equal efficiency.

Platinum(II) complexes, long central to chemotherapy, now exploit pH‐responsive self‐assembly for precision delivery [145, 146, 147]. Complexes **82–84** (Figure 14b), bearing pyrazole‐based pincer ligands, form orange phosphorescent aggregates in acidic lysosomes [97]. This self‐assembly induces lysosomal membrane permeabilization and cell death, with additional hydrogel formation enabling sustained drug release. The accompanying emission color shift (orange → green) offers real‐time visualization of drug activation.

Mixed‐metal complexes combine complementary properties into single scaffolds [148, 149, 150]. Heterobimetallic iridium(III)‐rhenium(I) complexes **71–73** (Figure 12) display strong emission enhancement at lysosomal pH (∼2.4) with cytotoxicity surpassing cisplatin [85]. These complexes trigger apoptosis via mitochondrial ROS pathways, inhibit migration, and induce protective autophagy, offering a multifaceted therapeutic profile.

Overall, pH‐responsive zinc(II), ruthenium(II), iridium(III), platinum(II), and mixed‐metal complexes illustrate how tumor acidity can be exploited for precise, multifunctional therapy. By combining selective activation with diagnostic luminescence, these systems point toward a promising strategy for personalized cancer theranostics.

## 4 Conclusion and Perspectives

pH‐sensitive luminescent transition metal complexes have proven to be remarkably versatile, with applications ranging from sensing and bioimaging to cancer‐related therapies. Their unique ability to respond to pH arises from reversible protonation and deprotonation, which subtly alter the electronic structure and excited‐state behavior, producing detectable changes in luminescence. Researchers have explored a wide variety of metals, including zinc(II), ruthenium(II), iridium(III), and platinum(II), and have employed clever molecular strategies such as H‐bonding, secondary‐sphere interactions, and aggregation‐induced emission to fine‐tune their responses.

Despite these advances, challenges remain. Ensuring long‐term stability, fully reversible behavior, and reliable signals in complex biological environments is still difficult. A deeper understanding of how ligand design, coordination geometry, and secondary interactions affect excited‐state behavior will be critical to overcoming these limitations.

Looking forward, several directions are particularly promising. Improving sensitivity and reversibility across biologically relevant pH ranges will require precise control over protonation sites and molecular motifs. Expanding beyond precious metals to include earth‐abundant and biocompatible metals such as iron(II), copper(I), and zinc(II) could enhance sustainability, provided their reactivity and toxicity are carefully managed. Multifunctional systems that combine pH sensing with therapeutic actions, such as photodynamic or photothermal effects, are especially attractive. Incorporating nanostructured or supramolecular designs can further improve solubility, cellular uptake, and *in vivo* performance, bringing these complexes closer to practical applications.

With ongoing rational design and a deeper understanding of their mechanisms, pH‐sensitive luminescent metal complexes are set to become even more reliable, effective, and practical tools for sensing, imaging, and therapeutic use.

## Conflicts of Interest

There is no conflict to declare.
